# Fifth Lumbar L5 Perineural Cyst with Unusual Radiculopathy: Traction Plexopathy

**DOI:** 10.7759/cureus.2052

**Published:** 2018-01-11

**Authors:** Tarush Rustagi, Swamy Kurra, William Lavelle

**Affiliations:** 1 Department of Spine Surgery, Indian Spinal Injuries Center, New Delhi, IND; 2 Department of Orthopedic Surgery, State University of New York Upstate Medical University, Syracuse, USA

**Keywords:** perineural cyst, tarlov cyst, radiculopathy, nerve sheath, traction plexopathy

## Abstract

Perineural cysts are cystic dilations and are frequently seen in relation to the lumbosacral spine. We describe a case of a fifth lumbar (L5) perineural cyst with unusual radicular symptoms and discuss the possible role of traction plexopathy caused by the cyst. A 38-year-old male presented with a longstanding history of back pain and right side thigh pain. This pain radiated from the buttocks to the lateral and anterior aspect of the thigh. He described the pain as pins and needles/burning with no significant relief with medications or rest. Imaging of the lumbar spine revealed a cystic lesion on the right side involving the L5 nerve root in the foraminal region. He failed conservative treatment and elected to have the cyst removed even with a guarded prognosis. A wide L5 laminectomy was performed. Due to the size of the cyst which was causing traction on the exiting L5 nerve root, the L5 pedicle was excised in order to delineate the cyst and to prevent any iatrogenic injury to the root. The patient had the dramatic improvement in his radicular pain immediately after the surgery and continues to be pain-free at his latest three-year follow-up. This case highlights the unusual pain pattern distribution from a perineural cyst possibly secondary to traction effect of the tumor.

## Introduction

Sciatic pain is a common condition most often attributed to lumbar degenerative disc disease. Other rare causes of compressive intra-spinal sciatica include the tumors of the nerve sheath. Tarlov cysts, also known as perineural cysts, are dilations of the nerve root sheath and filled with cerebrospinal fluid (CSF) and are usually located in the sacrum. These cysts can cause painful radiculopathy. The perineural cysts are cystic dilations and are frequently seen in relation to the lumbosacral spine. We describe a case of an L5 perineural cyst with unusual radicular symptoms and discuss the possible role of traction plexopathy caused by the cyst.

## Case presentation

**Figure 1:**
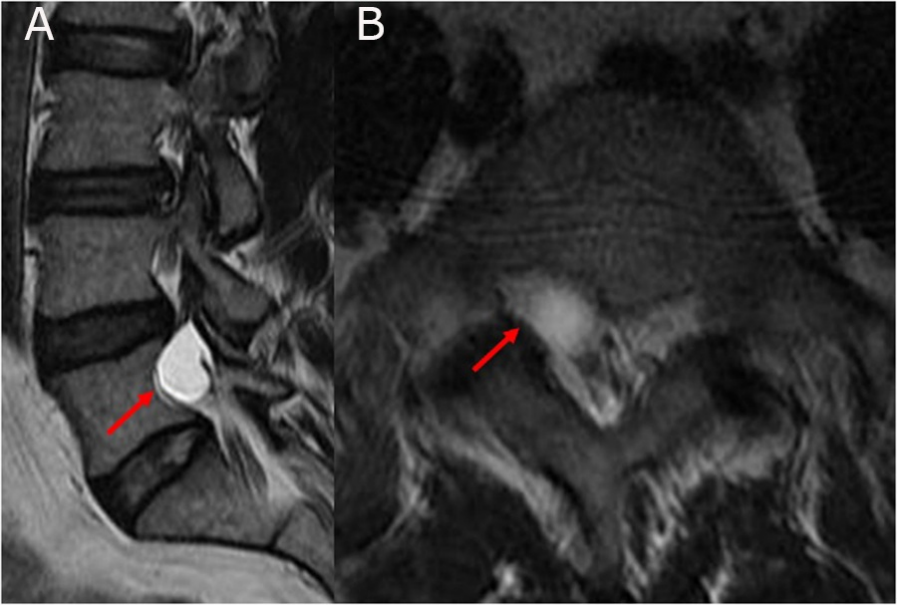
A) The right parasagittal T2W image showing fluid-filled cyst involving the fifth lumbar (L5) nerve root (red arrow). B) The axial T2W image showing the foraminal location of the cyst (red arrow). Note: The image quality is poor because the patient was unable to lie still during the magnetic resonance imaging (MRI).

**Figure 2:**
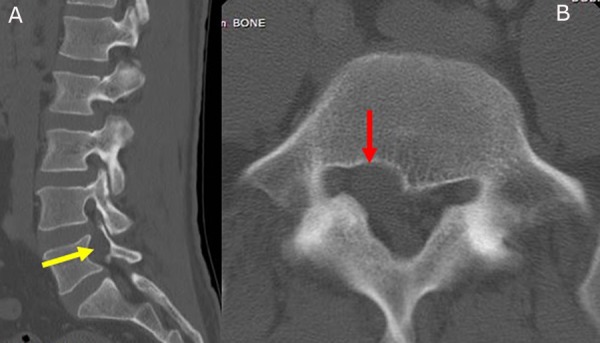
A) The right parasagittal computed tomography (CT) image showing the expansion of the neural foramen (yellow arrow). B) The axial CT scan showing the mass effect of the cyst resulting in the bone resorption and enlargement of the foramen (red arrow).

A 38-year-old male presented with a longstanding history of back pain and right side thigh pain. This pain radiated from the buttocks to the lateral and anterior aspect of the thigh. He described the pain as pins and needles/burning with no significant relief with medications or rest. On presentation, the pain had progressed to the state where it was interfering with his sleep. The magnetic resonance imaging (MRI) of the lumbar spine revealed a cystic lesion on the right side involving the fifth lumbar (L5) nerve root in the foraminal region (Figure 1). A computed tomography (CT) scan of the lumbar spine revealed a mass effect from the cyst resulting in bone resorption and widening of the L5 foramen (Figure 2). There was no complaint of any pain below the knee. He failed the conservative treatment, including a trial of image-guided steroid injection. After discussion, the patient elected to have the cyst removed even with a guarded prognosis. 

**Figure 3:**
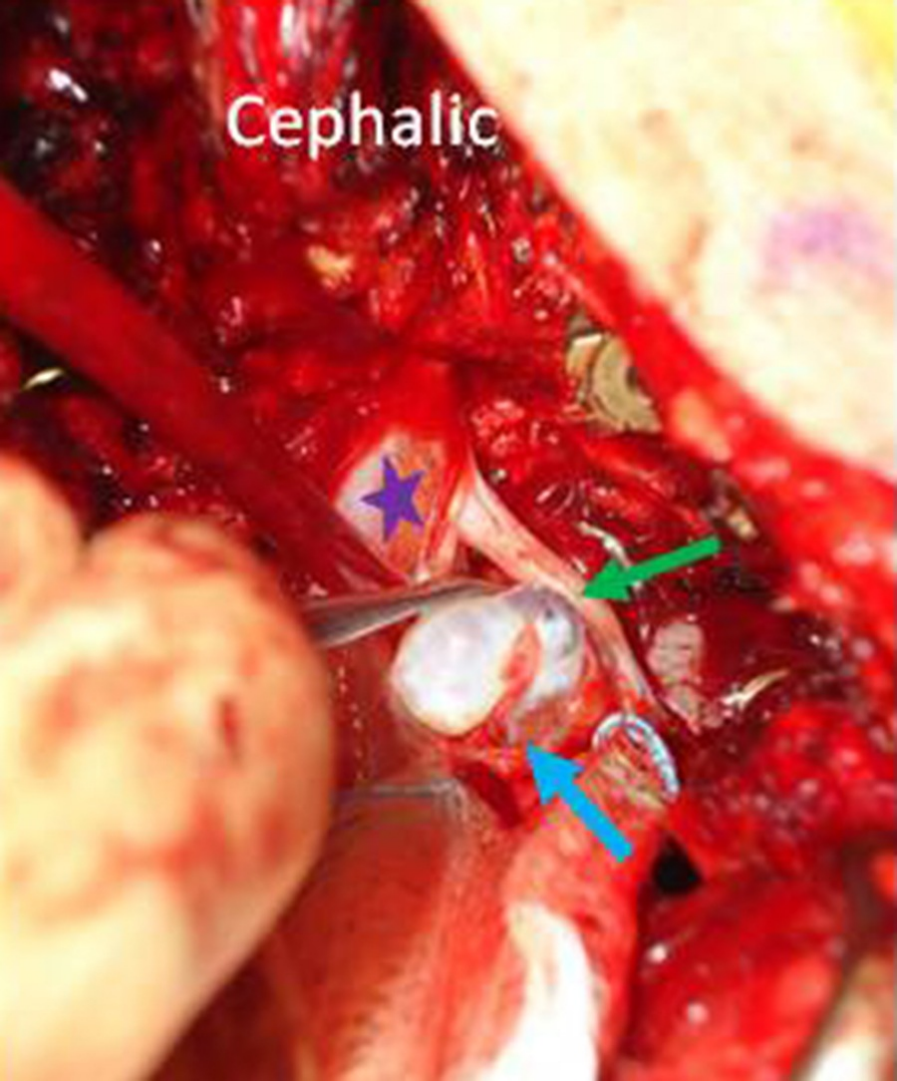
The perineural cyst (blue arrow) being separated from the fifth lumbar (L5) exiting root after tying off the neck. The exiting L5 nerve root (green arrow) and thecal sac (purple star).

**Figure 4:**
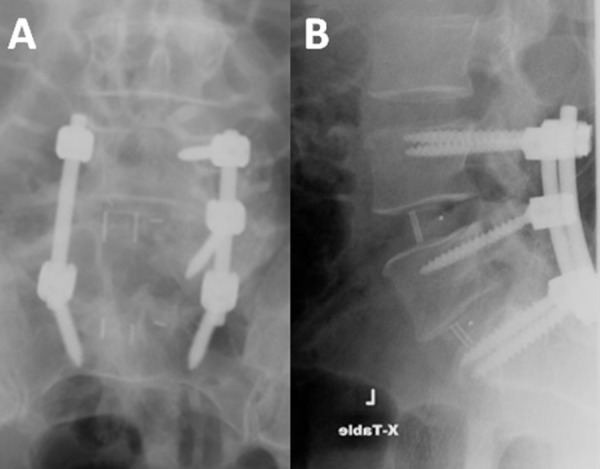
A) The postoperative image showing the right fifth lumbar (L5) pedicle excision. B) The postoperative image showing lumbar four and sacrum one (L4-S1) interbody fusion.

A wide L5 laminectomy was performed. Due to the size of the cyst which was causing traction on the exiting L5 nerve root, the L5 pedicle was excised in order to delineate the cyst and to prevent any iatrogenic injury to the root. The cyst was located in the axilla (cyst lying in the angle made by the thecal sac and the exiting L5 root) of the L5 nerve root. The cyst was meticulously separated and was found to be attached to the axilla through a narrow neck (Figure 3). Silk ties were used to tie off the neck and the cyst was excised in toto. The histopathology confirmed the perineural cyst. Instrumented fusion was completed from lumbar four to the sacrum one (L4-S1) (Figure 4).

The patient had the dramatic improvement in his radicular pain immediately after the surgery. During his last follow-up of three years, he continued to be pain-free.

## Discussion

This is a reported case of a perineural cyst in close proximity to the L5 nerve root. The foraminal tumors have been found to be 1% - 5% where schwannomas are most common [1]. These tumors typically do not have a neural component and are symptomatic of the mass effect and can usually be excised safely [1]. The perineural cysts are a cystic dilation of the nerve root and are commonly seen in the sacrum [2]. In the sacrum, less than 1% of the cysts are symptomatic [2]. The authors have reported a similar case of an L5 foraminal schwannoma that presented with pain and weakness along the corresponding L5 nerve root that responded to the surgical treatment [3].

This present case had an unusual clinical presentation. The patient was examined thoroughly for his intractable anterior thigh pain that did not match with the location of the cyst at L5. We investigated our case with electromyography/conduction studies that were inconclusive of any entrapment neuropathy. The MRI imaging of the pelvis failed to identify any intra-pelvic cause of the pain. The diagnosis injections were administered to rule out meralgia paraesthetica; and in addition, a diagnostic left side transforaminal block was not helpful. We also did a psychological evaluation of the patient to rule out a somatic disorder. We were unable to determine why the patient had anterior thigh pain which was eventually relieved after excision of the cyst.

One possible reason for the anterior thigh pain may be a traction effect of the nerve roots caused by the cyst which was enclosed into the foramen. The traction plexopathy is a well-known phenomenon reported in relation to the brachial plexus [4-5].

One may correlate a similar instance of a traction phenomenon that often requires the surgery in tethered cord syndrome, where the pull is exerted onto the spinal cord from a persistent tethered cord [6]. While it is difficult to explain the nature of the pull in our case, we believe the proximal root might have been pulled by the anchored and gradually expanding nature of the neural cyst. This might have caused a traction effect over the proximal lumbar three and four (L3 and L4) roots and the reason why the patient did not get any relief from an image-guided transforaminal injection of the L5 nerve root.

Numerous techniques have been described for the treatment. The surgeries needing a shunt are based on the equalization of the CSF pressure with the dural sac [7-8], more radical surgeries include sacrificing the parent root [9]. The percutaneous cyst drainage has also been described as a treatment option with variable outcomes [10]. Excision of the cyst is possible in most cases and is known to result in close to 100% pain relief in most patients [2]. We chose to do the excision of the perineural cyst.

Most of the cases of perineural cysts are described in the sacral spine where stability is not a major concern following a laminectomy. In our case, the cyst was in close proximity to the pedicle, and we found it unsafe to completely delineate and excise it without removing the pedicle; thus, making fusion necessary.

## Conclusions

Our patient had complete resolution of the radiating pain, including the anterior thigh. In our case, we highlighted the unusual pain pattern distribution from a perineural cyst possibly secondary to a traction effect of the tumor.
